# Performance Analysis of Serodiagnostic Tests to Characterize the Incline and Decline of the Individual Humoral Immune Response in COVID-19 Patients: Impact on Diagnostic Management

**DOI:** 10.3390/v16010091

**Published:** 2024-01-06

**Authors:** Ronald von Possel, Babett Menge, Christina Deschermeier, Carlos Fritzsche, Christoph Hemmer, Hilte Geerdes-Fenge, Micha Loebermann, Anette Schulz, Erik Lattwein, Katja Steinhagen, Ralf Tönnies, Reiner Ahrendt, Petra Emmerich

**Affiliations:** 1 Department for Virology, Bernhard Nocht Institute for Tropical Medicine, 20359 Hamburg, Germany; 2 Department of Tropical Medicine and Infectious Diseases, Center of Internal Medicine II, University of Rostock, 18057 Rostock, Germany; 3 Institute for Experimental Immunology, EUROIMMUN Medizinische Labordiagnostika AG, 23560 Lübeck, Germany; 4 Diagnostics Development Laboratory, Bernhard Nocht Institute for Tropical Medicine, 20359 Hamburg, Germany; 5 medac GmbH, 22880 Wedel, Germany

**Keywords:** COVID-19, individual humoral immune response, serodiagnostic tests

## Abstract

Serodiagnostic tests for antibody detection to estimate the immunoprotective status regarding SARS-CoV-2 support diagnostic management. This study aimed to investigate the performance of serological assays for COVID-19 and elaborate on test-specific characteristics. Sequential samples (*n* = 636) of four panels (acute COVID-19, convalescent COVID-19 (partly vaccinated post-infection), pre-pandemic, and cross-reactive) were tested for IgG by indirect immunofluorescence test (IIFT) and EUROIMMUN EUROLINE Anti-SARS-CoV-2 Profile (IgG). Neutralizing antibodies were determined by a virus neutralization test (VNT) and two surrogate neutralization tests (sVNT, GenScript cPass, and EUROIMMUN SARS-CoV-2 NeutraLISA). Analysis of the acute and convalescent panels revealed high positive (78.3% and 91.6%) and negative (91.6%) agreement between IIFT and Profile IgG. The sVNTs revealed differences in their positive (cPass: 89.4% and 97.0%, NeutraLISA: 71.5% and 72.1%) and negative agreement with VNT (cPass: 92.3% and 50.0%, NeutraLISA: 95.1% and 92.5%) at a diagnostic specificity of 100% for all tests. The cPass showed higher inhibition rates than NeutraLISA at VNT titers below 1:640. Cross-reactivities were only found by cPass (57.1%). Serodiagnostic tests, which showed substantial agreement and fast runtime, could provide alternatives for cell-based assays. The findings of this study suggest that careful interpretation of serodiagnostic results obtained at different times after SARS-CoV-2 antigen exposure is crucial to support decision-making in diagnostic management.

## 1. Introduction

Severe acute respiratory syndrome coronavirus 2 (SARS-CoV-2) causes coronavirus disease-19 (COVID-19) [1,2,3] with mild, severe, or fatal courses. Due to the rapid global spread of SARS-CoV-2, the World Health Organization declared a pandemic in March 2020 [4,5]. Along with the human coronaviruses (hCoV) HKU1 and OC43, SARS-CoV-2, like SARS-CoV and MERS-CoV, belongs to the beta-CoVs. Additionally, alpha-CoVs 229E and NL63 are found worldwide. CoVs typically cause mild upper respiratory disease [4].

The entry of SARS-CoV-2 into cells is mainly mediated by the interaction of the homotrimeric viral spike (S) glycoprotein with the human angiotensin-converting enzyme 2 (hACE2) on host cells [6]. The S protein is composed of two subunits, S1 and S2 [7], with the receptor binding domain (RBD) located on S1. Following the binding of S1/RBD to hACE2, S1 and S2 are cleaved by host proteases, which induce the activation of S2. Activated S2 mediates the fusion of the viral particle with the cellular membrane [6]. This fusion results in the release of viral RNA, which is encapsulated by the nucleocapsid (N) protein, for virus replication [8]. N is an abundantly produced viral protein [9] and serves multiple functions, such as the regulation of viral replication and RNA assembly [8].

Nucleic acid amplification tests, such as Real-Time Reverse Transcription Polymerase Chain Reaction (rRT-PCR), are used to detect acute SARS-CoV-2 infections [10,11]. Analysis of the host immune response can indirectly identify individuals with a current or past infection and monitor vaccine efficacy [12,13]. Indirect immunofluorescence tests (IIFT) can detect seroconversion by determining immunoglobulin (Ig) classes IgG, IgM, and IgA antibodies (Ab) in response to the full antigenic spectrum of SARS-CoV-2-infected cells [14]. Virus neutralization tests (VNT) detect the inhibition of viral infection by neutralizing antibodies (NAb) present in patient serum [15]. These cell culture-based assays serve as the gold standard even though they are time-consuming, labor-intensive, and require a biosafety level 3 (BSL3) laboratory [16]. Another disadvantage is their unsuitability for large-scale use and the lack of inter-laboratory standardization [15]. Commercially available technologies such as enzyme-linked immunosorbent assays (ELISA) and immunoblots that specifically detect Abs targeting the main SARS-CoV-2 immunogens S1/RBD, S2, and N [17,18] are potential alternatives for cell-based serological assays. They are less time-consuming, do not require a BSL3 environment, and improve comparability between laboratories. Surrogate virus neutralization assays (sVNT) detect the binding between recombinant hACE2 and recombinant viral RBD in an ELISA format with a colorimetric readout [19]. The color intensity is inversely proportional to the NAb concentration in the sample.

Several studies have investigated commercially available S1/RBD-based assays, mainly analyzing a single sample per patient [16,20,21,22,23]. In this study, the performance of a line blot was compared with that of an IIFT for the detection of SARS-CoV-2-specific IgG and of two sVNTs with a VNT for the detection of NAbs, thereby focusing primarily on sequential sera at different phases of COVID-19 without and with vaccination. By doing so, this study aimed to achieve a better understanding of the performance of the different assays in different stages of COVID-19 and after vaccination and how this affects their interpretation.

## 2. Materials and Methods

Patient data are summarized in Table 1. Panel A consists of 268 sequential and single samples obtained from 115 patients with mild to severe COVID-19 symptoms over a total period of 0–154 days post-symptom onset (dpso) between March 2020 and April 2021. SARS-CoV-2 was confirmed by real-time RT-PCR (Allplex 2019-nCoV Assay, Seegene Inc., Seoul, Republic of Korea). The panel comprises 15 patient samples with the following comorbidities: cancer (*n* = 10), immunosuppression after organ transplantation (*n* = 3), and ongoing Ab therapy (*n* = 4), including 2 cancer patients. Panel B contains pre-pandemic sera from 95 blood donors obtained before August 2019. Panel C is composed of 237 sequential and single samples from 36 convalescent COVID-19 patients (PCR [24] confirmed) with mild symptoms. The samples were obtained between March 2020 and March 2021 and cover a period of combined 10 to 474 dpso. Of those patients, 21 were vaccinated within the time of this study using the vaccines Ad26.COV2.S, BNT162b2, mRNA-1273, AZD122, or combination schedules of these vaccines. Panel D contains sera from patients with seasonal hCoV infection (*n* = 12) and rhinovirus infection (*n* = 1) obtained between January 2020 and April 2020. The infection was confirmed by IIFT [13].

The detection of SARS-CoV-2-specific IgG was performed by means of IIFT as previously described [25] using cells infected with the SARS-CoV-2 patient isolate HH-1 [26]. Briefly, SARS-CoV-2-infected VeroE6 cells (ATCC CRL-1008) were fixed with acetone-methanol and incubated with diluted (1:10 to 1:80) patient sera. Human IgG was detected using an anti-human IgG fluorescein isothiocyanate conjugate. Specimens were analyzed with a fluorescence microscope. Titers below 1:20 were considered negative.

Samples were additionally analyzed using the EUROLINE Anti-SARS-CoV-2 Profile (IgG) (EUROIMMUN, Medizinische Labordiagnostik AG, Lübeck, Germany). The Profile IgG is a line blot coated with purified S1, S2, and N of SARS-CoV-2 (Wuhan-Hu-1), as well as N of seasonal hCoVs, for the determination of the corresponding IgG in serum or plasma. The assay was performed according to the manufacturer’s instructions with diluted (1:51) serum. Samples were classified as positive (≥18), equivocal (12–18), or negative (≤11) based on line intensities for each antigen measured using the EUROLineScan software 3.4.32 (EUROIMMUN). A sample was anti-SARS-CoV-2 positive when at least two of the three line intensities (S1, S2, and N of SARS-CoV-2) were above the cutoff. It was considered equivocal if S1 or N were positive, and at least one further band was equivocal. If none of this applied, the sample was negative.

The VNT for the detection of NAbs was performed according to Brehm et al. [26]. Heat-inactivated (56 °C, 30 min) serially diluted patient sera (1:20 to 1:5120, triplicates) were mixed with an equal volume of viral solution containing 20 tissue culture infectious dose 50 (TCID_50_) of SARS-CoV-2 (patient isolate HH-1) and transferred to VeroE6 cells, seeded the previous day with a concentration of 5 × 10^6^ per well of a 96-well plate. Cells were incubated at 37 °C with 5% CO_2_ in a humidified environment for 96 h. The supernatant was discarded, and cells that were not detached by the cytopathic effect (CPE) were fixed with 4% formaldehyde and stained with crystal violet for CPE evaluation. The highest dilution protecting cells from CPE in two of three wells was assessed as NAb titer. A titer of 1:40 was used as a cutoff.

In addition to the studied VNT, two different surrogate virus neutralization tests, the GenScript cPass (GenScript, Piscataway Township, NJ, USA) and the SARS-CoV-2 NeutraLISA (EUROIMMUN), were used to determine NAb. The tests were performed according to the instructions of the respective manufacturer. Briefly, the cPass was performed using diluted serum (1:10) preincubated with horseradish peroxidase (HRP)-labeled RBD (37 °C, 30 min) and transferred to hACE2-precoated wells (37 °C for 15 min). RBD and hACE2 binding were detected by HRP-catalyzed color reactions. A signal strength of ≥30% was considered positive, and <30% was negative. 

The NeutraLISA was performed using serum samples diluted (1:5) with sample buffer containing biotinylated hACE2 and added to RBD-precoated wells (37 °C, 60 min). HRP-labeled streptavidin was added (30 min), followed by detection using a chromogen and absorbance measurement. Samples were evaluated as positive, equivocal, and negative if the inhibition rate was ≥35%, between 20% and 35%, and ≤20%, respectively.

Inter-assay concordances were calculated as percentages of agreement with Clopper-Pearson confidence intervals (CI). Equivocal results (EUROLINE Profile IgG and EUROIMMUN SARS-CoV-2 NeutraLISA) were counted as positive. Statistical analysis was performed using the Wilcoxon rank sum test (GraphPad Prism 9) with Bonferroni correction.

## 3. Results

### 3.1. Assessment of Humoral Responses in Acute COVID-19 Using Different Assays

The development of the humoral immune response during acute COVID-19 was analyzed in samples from panel A (Table 1), irrespective of the severity of symptoms and comorbidities. Panel B samples served as a negative control.

Samples were categorized according to their time of collection (dpso/dpPCR) into T1 (0–5), T2 (6–10), T3 (11–20), and T4 (>20) and tested for IgG seroconversion (Table 2). In T1, anti-SARS-CoV-2-specific IgG was detected in 21.0% (13/62) of the samples by IIFT and in 8.1% (5/62) by the Profile IgG. As the infection progressed, the seropositivity increased to 61.3% (49/80) and 48.8% (39/80) in T2, as well as 80.0% (60/75) and 72.0% (54/75) in T3 using the IIFT and Profile IgG, respectively. In T4, IIFT and Profile IgG found 80.0% (16/20) and 85.0% (17/20) of samples positive, respectively (Supplementary Figure S1). Overall, SARS-CoV-2-specific IgG antibodies were detected in 57.1% (153/268) of patient samples by IIFT and in 47.0% (126/268) by Profile IgG. Calculations revealed a positive agreement of 78.4% and a negative agreement of 94.8% for the Profile IgG in relation to the results obtained by IIFT. No Abs were detected in the pre-pandemic samples using both tests, indicating a diagnostic specificity of 100% for the IIFT and Profile IgG.

Neutralizing activity was determined in the same panels using an in-house VNT in comparison with the sVNTs (cPass and NeutraLISA). In T1, the VNT revealed NAbs in 11.3% (7/62) of sera, the cPass determined inhibition in 12.9% (8/62) and the NeutraLISA in 6.4% (2 positive plus 2 equivocal/62) (Table 2). With increasing dpso/dpPCR, the proportion of samples with NAb increased. In T2 as well as T3, the VNT, cPass, and NeutraLISA characterized 61.3% (49/80), 52.2% (42/80), and 35.1% (23 positive plus 5 equivocal/80) as well as 77.3% (58/75), 76.0% (57/75), and 65.3% (40 positive plus 9 equivocal/75) samples as positive, respectively. Remarkably, the cPass yielded positive results in a higher number of early samples compared to the NeutraLISA. In T4, all tests found 85.0% (17/20) of samples positive (15 positive plus 2 equivocal/20, NeutraLISA). Using the VNT as a reference, an overall positive agreement of 88.3% and a negative agreement of 92.7% for the cPass, as well as a positive agreement of 71.7% and a negative agreement of 95.9% for the NeutraLISA, were calculated. No tests detected NAbs in prepandemic sera, which indicates a diagnostic specificity of 100% for all assays.

The cohort of acute cases included patients diagnosed with cancer, treated with immunosuppressives after organ transplantation, or receiving SARS-CoV-2 antibody therapy (Supplementary Figure S2). One patient with follicular lymphoma, which affects the immune system, did not develop a humoral immune response within 30 dpso. On the other hand, a patient with Hodgkin’s lymphoma established IgG seroconversion and produced NAbs at levels comparable to patients without comorbidities. In two of three patients treated with immunosuppressives, seroconversion was detectable at 7 dpso/dpPCR. Noteworthy are the results from patients on SARS-CoV-2 therapy with S1-based antibodies. These samples were positive for NAb in all assays and for IgG in the IIFT as well as for anti-S1 IgG, yet overall negative in the Profile IgG.

### 3.2. Dynamics of the Humoral Immune Response in a Convalescent COVID-19 Patient Cohort

Sequential convalescent samples (*n* = 237) from 36 PCR-confirmed patients over a period of up to 474 days (Table 1, Panel C) were analyzed. Anti-SARS-CoV-2 IgG antibodies were detected in 100% using the IIFT, whereas the Profile IgG showed a positive result for 91.6% (216 positive plus 1 equivocal/237) of samples, corresponding to a positive agreement of 91.6% (Table 3).

In all patients, IgG antibody levels decreased or stagnated over time until another antigen exposure by re-infection (sample CoV24) or vaccination (Supplementary Figure S3). This second stimulus led to a stronger increase in IgG detected in both the IIFT and Profile IgG than in most cases of infection alone. Noteworthy, the vaccination induced a strong anti-S1/2 IgG but no anti-N IgG response.

Additionally, the dynamics of the NAbs were investigated using the VNT as well as both sVNTs (Table 3). Samples were grouped based on their collection times (dpso/dpPCR), as follows: T5 (0–150), T6 (151–300), T7 (301–500), as well as TVac (days post-vaccination).

**Figure 1:**
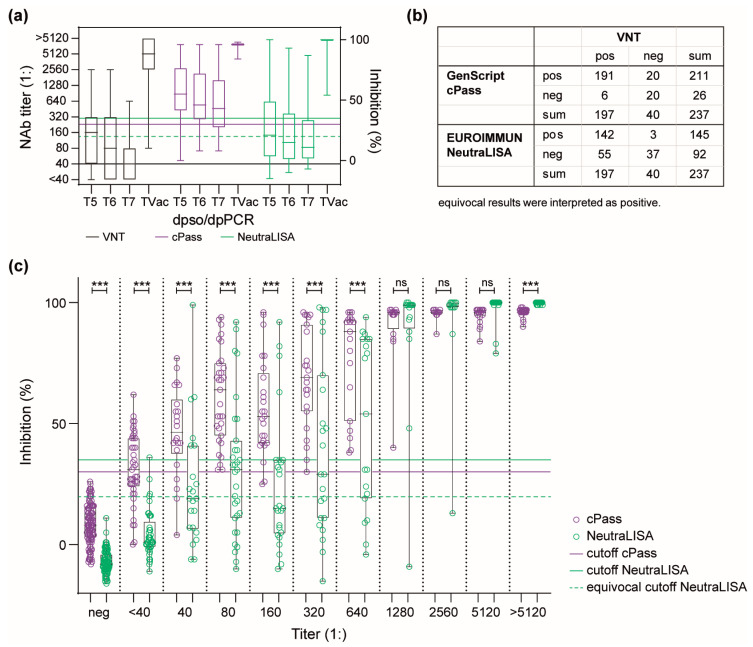
(**a**) Decrease of NAbs in sera from convalescent patients over time, as determined by three different NTs. Results obtained using VNT (black) are plotted on the left *Y*-axis; those obtained using cPass (purple) and NeutraLISA (green) are plotted on the right *Y*-axis. (**b**) Number of NAb positive and negative sera by cPass and NeutraLISA compared to VNT. (**c**) Comparison of inhibition detected by cPass and NeutraLISA at the corresponding VNT NAb titers: *** = *p* ≤ 0.001, ns = not significant, and neg = pre-pandemic samples.

Over time, the median NAb titers decreased from 1:160 (T5) to 1:40 (T7). This decrease was also seen by cPass and NeutraLISA, as reflected by median inhibition rates declining from 57.5% to 42.8% and from 21.2% to 10.8%, respectively (Figure 1A). Similarly, a decline in maximum values from T5 to T7 for the VNT (titers of 1:2560 to 1:640) and the NeutraLISA (inhibition of 100% to 87%) was noticed. In contrast, no relevant titer change was observed for the cPass (96% for T5 and T7).

The inhibition values detected with both sVNTs correlated with the corresponding titers determined by the VNT, as indicated by Spearman’s rank correlation coefficients (cPass: r = 0.83 [95% CI: 0.78–0.86], NeutraLISA: r = 0.79 [95% CI: 0.74–0.84]). However, the assays differed in the number of NAb-positive and negative samples. In T5, the VNT detected NAbs in 81.8% (81/99) of sera, the cPass in 87.9% (87/99), and the NeutraLISA in 51.5% (35 positive plus 16 equivocal/99). In T6, 73.3% (33/45), 84.4% (38/45) and 40.0% (11 positive plus 7 equivocal/45) and in T7, 60.0% (15/25), 72.0% (18/25) and 32.0% (6 positive plus 2 equivocal/25) of samples were found NAb positive by the VNT, cPass, and NeutraLISA, respectively. Noteworthy, in all intervals, the cPass detected more NAb-positive samples than the VNT and the NeutraLISA. The TVac subpanel consists of sequential samples from 21 patients taken after vaccination or re-infection. During this period, the highest NAb titers (≥1:1280) were found in 94.1% (64/68) of sera using the VNT. Similarly, the highest inhibition values (75% to 100%) were detected in 100% (68/68) and 98.4% (67/68) by cPass and NeutraLISA, respectively. The dynamics of SARS-CoV-2-specific IgG and NAb are shown in Supplementary Figure S4, and the course of NAbs in all single samples is shown in Supplementary Figure S5.

Overall, based on the samples from convalescent patients and related to the VNT, there was a positive agreement of 97.0% and a negative agreement of 50.0% for the cPass, as well as a positive agreement of 72.1% and a negative agreement of 92.5% for the NeutraLISA. Although both sVNTs are based on the inhibition of binding between S1/RBD and ACE2, there were numerous differences in measured inhibition values and hence NAb-positive or negative evaluation (Figure 1B). Therefore, the medians of inhibition rates determined by sVNTs at different NAb titers were compared. A significant difference (*p* < 0.001) was found at all titers ≤1:640. Here, the inhibition values determined by cPass were on average 2.4 times higher than those obtained by NeutraLISA, except for values at titers <1:40, where measured values were 7.2 times higher. In samples of the negative cohort, there was a median difference of 16% (Figure 1C). At titers ≥1:2560, inhibition rates reached saturation in both sVNTs. Only at titers > 1:5120, there was a marginal (1.04-fold) but significantly higher inhibition rate with NeutraLISA.

Panel C included one patient with SARS-CoV-2 reinfection (sample CoV24, Supplementary Figure S3) [26]. At day one pso of the reinfection, the detected NAb titer was 1:80 and increased to 1:1280 at 16 dpso. At the same time, the inhibition rate detected by NeutraLISA increased similarly, from −10% to 99%. However, the increase in inhibition seen by cPass was less dramatic (84% to 96%). Interestingly, the cPass detected an increase in inhibition from 74% (75 days before reinfection) to 84% (one day post-reinfection), whereas a decrease from 33% to −10% was seen by the NeutraLISA and a stagnation of NAb titer at 1:80 (VNT) during the same time.

### 3.3. Performance of Antibody Detection in Patient Samples after Infection with Seasonal hCoVs

**Figure 2:**
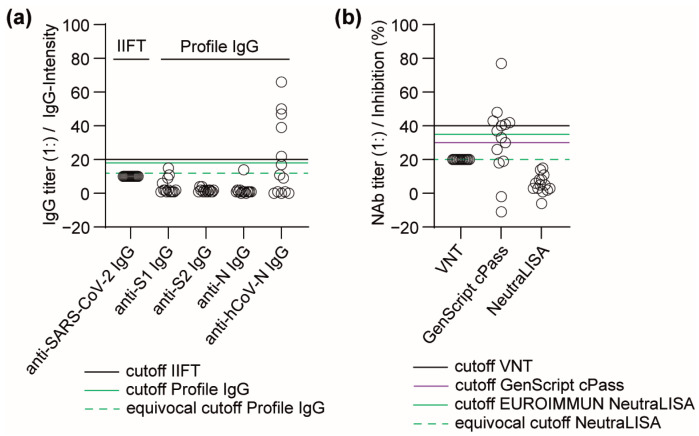
Analytical specificity of (**a**) Profile IgG and (**b**) cPass and NeutraLISA in sera from patients with seasonal hCoV infection.

As structural similarities between SARS-CoV-2 and other seasonal hCoV may affect the analytical specificity of a serodiagnostic test, cross-reactivities were studied in a cohort comprising pediatric (*n* = 10) and adult sera (*n* = 4) from patients with seasonal hCoV infections (*n* = 12) or rhinovirus infection (*n* = 1). No SARS-CoV-2-positive IgG was detected by the IIFT or Profile IgG. Anti-N IgG against the respective seasonal hCoV was found in 6 out of 14 sera, but not in the sample with rhinovirus infection. SARS-CoV-2-specific NAb were neither detected by VNT nor by NeutraLISA. However, the cPass detected SARS-CoV-2-specific NAbs in 8/10 pediatric and 0/4 adult sera (Figure 2).

## 4. Discussion

As COVID-19 is a crisis affecting public health and the global economy [27], it is important to know the immune status of individuals for planning vaccination campaigns [28] as well as for implementing healthcare and economic measures [27]. Here, the results obtained by different diagnostic tests were compared, and the tests’ analytical performance in the determination of IgG and NAb during acute (panel A) and convalescent (panel C) COVID-19 infections as well as in pre-pandemic (panel B) and seasonal hCoV infection (non-SARS-CoV-2 infection, panel D) cohorts was assessed.

The positive agreement of the results of the Profile IgG with those of the IIFT is high. Nevertheless, it increases from panel A (78.3%) to panel C (91.6%). This could be due to different test principles and the manufacturer’s instructions on how to interpret the results. In contrast to the IIFT, which contains the full spectrum of viral antigens in the correct conformation, the Profile IgG comprises immobilized S1, S2, and N as antigens, which are immunodominant yet limited [17,29]. As more epitopes are available in the IIFT, its sensitivity might be generally higher. Additionally, according to the instructions for the interpretation of Profile IgG results, a sample is positive if at least two of the three antigen bands show intensities above the cutoff. N of CoVs induces an antibody response earlier than S [17,30,31]. This potentially contributes to the lower positive agreement in Panel A. Seroconversion of IgG is mainly observed between one and three weeks after symptom onset [32]. After this time, SARS-CoV-2-specific Abs were detected with both assays, as reflected by the high agreement in Panel C. However, the decrease of Abs against CoV antigens occurs at different times in different individuals.

Different results between the IIFT and Profile IgG were observed in patients receiving S1-based immunoglobulin therapy. Although the highest values for S1 were obtained with the Profile IgG, the samples were overall negative due to the instructions for result interpretation mentioned above but positive with the IIFT.

Immobilized N of seasonal hCoVs on the Profile IgG provides additional information on past CoV infections and could also be useful for cross-reactivity screenings [25,33] in vaccine studies, as novel N-based vaccines have been suggested [34,35].

Although IgG and NAb levels tend to correlate well [15], IgG-positive patients may be NAb-negative [22,36]. Thus, the determination of NAbs is probably a better predictor of the immune protection of a person [37] than simple IgG measurement. In the present study, the neutralizing activity of samples from the four different panels was tested with the cell culture-based VNT and two sVNTs, cPass and NeutraLISA. As NAbs are usually detected later than non-neutralizing IgG [38,39,40], the early detection of NAbs in T1 (0–5 dpso/dpPCR) by all three NTs was surprising. As dpso and dpPCR were equalized in this study and samples from panel A were analyzed irrespective of disease severity, the detectability of NAbs might shift to earlier times. By revising the data, two sera in T1 were found to originate from a patient receiving Ab therapy. However, in T1, the cPass yielded the same number of positive samples as the VNT and one false positive. Very early detection of SARS-CoV-2-specific Abs in acute COVID-19 and false-positive reactivity in pre-pandemic samples by the cPass have been previously described [23,41], and it has been speculated that non-neutralizing Abs were also recognized [22]. Here, no false-positive samples were found in panel B. Nevertheless, the inhibition rates seen in the cPass were closer to the cutoff levels than the inhibition rates seen in the NeutraLISA. However, the detection of non-specific Abs could explain the different course of NAbs in the CoV24 sample found by cPass compared to NeutraLISA and VNT.

The higher positive agreement (97.0%) with VNT at a lower negative agreement (50.0%) for the cPass and vice versa for the NeutraLISA (71.6% and 92.5%) in the convalescent sera are in line with previous studies [20,23,42]. Interestingly, the cPass had a lower negative agreement in panel C (50.0%) compared to panel A (92.3%), while the NeutraLISA showed similar agreements (92.5% and 95.2%). The difference in agreements is striking, as both tests are based on the inhibition of binding between RBD of SARS-CoV-2 and hACE2. In the cPass, the preincubation of serum with HRP-labeled RBD might enhance the assay’s sensitivity but could also lead to overestimation of the neutralizing activity [43,44]. The competitive binding of serum antibodies and biotinylated hACE2 to coated RBD in the NeutraLISA could decrease the assay’s sensitivity and thus lead to more false-negative samples [23,42], even though competitive binding might reflect the in vivo situation. Although most NAbs are directed against the S1/RBD domain (the detected antigen in sVNTs), NAbs against other epitopes were described [45]. Thus, the false-negative results of sVNTs could be attributed to the broader antigenic spectrum of cell culture-based NTs. Since both sVNTs correlate strongly with the VNT, they should also yield a negative result for these samples.

Despite the correlation, higher inhibition rates by cPass compared to those obtained by NeutraLISA in almost all sera, excluding post-vaccination samples, were observed, which was similarly shown by others without emphasizing this fact [16,23,46]. The higher inhibition rates in cPass may be caused by the different assay setup. However, they were also shown in a comparative study of assays with the same underlying principle [43].

When investigating the cross-reactivity of sVNTs in a small cohort, SARS-CoV-2-specific NAbs were observed in stored pediatric samples (8 of 10) from patients with seasonal hCoV infection but not in adult samples (0 of 4) using cPass. No cross-reactivity was found using NeutraLISA. Interestingly, patients with syphilis infections but not with seasonal hCoV infections were reported to be positive for cPass [47,48]. This false-positive detection might be caused by preserving agents such as sodium azide in stored samples, as azide inhibits peroxidase activity [49]. Due to the different test setups, this inhibition impacts the cPass but not the NeutraLISA, although in both tests HRP catalyzes the color reaction. This makes NeutraLISA more robust for sample additives.

The data presented in this study are based on sera obtained between March 2020 and April 2021 from persons infected with the variants of SARS-CoV-2 circulating at that point. This is presumably the biggest drawback of this study. Many variants of concern (VOCs) have evolved since then. Since mutations mainly occur in S, they cause a substantial limitation in the detection ability of S1/RBD-based serological assays [23,50]. Assuming a similar immune response to S1 regardless of VOC, times of increase and decrease of NAbs after a COVID-19 infection should be similar and could be estimated, as well as conclusions regarding the immunoprotective status of an individuum drawn.

## 5. Conclusions

For serodiagnostics, the Profile IgG can be used as a fast alternative to cell culture-based IIFT to confirm the immune responses to COVID-19 infection and vaccination, starting approximately two weeks after the immunologic event. The immunoprotective function of Abs can be best determined by cell culture-based virus infection assays containing the full viral antigenic spectrum. However, as infection assays are restricted to BSL3 diagnostic labs and require a runtime of several days, depending on the procedure, sVNTs represent a less restricted alternative. The sVNTs studied here should be used for different purposes, as they differ in their positive and negative agreements with VNT. After adapting S1/RBD to the currently circulating VOCs, an early and low immune response can be determined by the cPass due to its high positive agreement, whereas the NeutraLISA is helpful to monitor waning NAbs, especially after vaccination, to support decision-making about booster shots as well as in patients receiving Ab therapy. All assays should be interpreted carefully, and the results should be complemented by the outcomes of other diagnostic tests. For instance, a combination of sVNT and a blot-based assay or IIFT could increase diagnostic accuracy, as more than one SARS-CoV-2-specific antigen would be used and test results would serve as internal controls.

## Figures and Tables

**Table 1:** The representation of cohorts used in this study.

	Panel A (Acute)	Panel B (Pre-Pandemic)	Panel C (Convalescent)	Panel D (Seasonal hCoV, Non-SARS-CoV-2)
Total number of patients	115	95	36	13
Age (mean ± SD, range) [years]	61.6 ± 18.6, 21–89	unknown	43.1 ± 13.4, 19–64	7.8 ± 4.2, 4–15
				75.7 ± 0.5, 75–89
Sex (*n* females, *n* males, *n* unknown)	58, 57, 0	unknown	24, 12, 0	3, 7, 0
				1, 2, 0
N samples in the panel	268	95	237	14
Number of sequential samples per patient	1–6	-	1–14	1–2
dpso/dpPCR (mean ± SD, range, unknown)	14.3 ± 20.6, 0–154, 31	-	199.1 ± 134.6, 10–474, 0	unknown
				68.7 ± 46.2, -, -
Total number of vaccinated patients in the cohort	-	-	21	-
N samples post-vaccination	-	-	63	-
Number of sequential samples post-vaccination	-	-	1–9	-
dpVac (mean ± SD, range)	-	-	52.1 ± 40.7, 6–181	-

Abbreviations: dpso: days post-symptom onset; dpPCR: days post-PCR; dpVac: days post-vaccination; SD: standard deviation.

**Table 2:** Comparative determination of SARS-CoV-2-specific IgG and NAb during acute COVID-19 infection (panel A) and in pre-pandemic samples (Panel B) by different diagnostic tests.

		Anti-SARS-CoV-2-IgG		Anti-SARS-CoV-2 NAb			
		In-House IIFT	EUROLINE Profile IgG	In-House VNT	GenScript cPass	EUROIMMUN SARS-CoV-2 NeutraLISA	
Period (dpso/dpPCR)	*N* total	*N* pos (%)	*N* pos (%)	*N* pos (%)	*N* pos (%)	*N* pos (%)	*N* equ (%)
T1 (0–5)	62	13 (21.0)	5 (8.1)	7 (11.3)	8 (12.9)	2 (3.2)	2 (3.2)
T2 (6–10)	80	49 (61.3)	39 (48.8)	49 (61.3)	42 (52.2)	23 (28.8)	5 (6.3)
T3 (11–20)	75	60 (80.0)	54 (72.0)	58 (77.3)	57 (76.0)	40 (53.3)	9 (12.0)
T4 (>20)	20	16 (80.0)	17 (85.0)	17 (85.0)	17 (85.0)	15 (75.0)	2 (10.0)
Unknown	31	15 (48.4)	11 (35.5)	14 (45.2)	13 (41.9)	11 (35.5)	2 (6.5)
All samples incl. unknown	268	153 (57.1)	126 (47.0)	145 (54.1)	137 (51.1)	91 (34.0)	20 (7.5)
Positive agreement % ^a^		reference	78.4	reference	88.3	71.7	
95% CI			71.1–84.7		81.9–93.0	63.7–78.9	
Negative agreement %		reference	94.8	reference	92.7	95.9	
95% CI			86.1–98.1		83.6–96.6	88.2–98.7	
Pre-pandemic sera	95	0	0	0	0	0	
Specificity % ^b^		100	100	100	100	100	
95% CI		96.2–100	96.2–100	96.2–100	96.2–100	96.2–100	

^a^ Calculation of agreements was based on the respective in-house tests, with equivocal results from EUROIMMUN SARS-CoV-2 NeutraLISA counted as positive. ^b^ Calculation of specificities was based on a clinical diagnosis. Abbreviations: pos, positive; equ, equivocal; dpso/dpPCR, days post-symptom onset/days post-PCR.

**Table 3:** Comparative determination of SARS-CoV-2-specific IgG and NAb in the convalescent COVID-19 cohort (panel C) by different diagnostic tests.

		Anti-SARS-CoV-2-IgG			Anti-SARS-CoV-2 NAb			
		In-House IIFT	EUROLINE Profile IgG		In-House VNT	GenScript cPass	EUROIMMUN SARS-CoV-2 NeutraLISA	
dpso/dpPCR	*N* total	*N* pos (%)	*N* pos (%)	*N* equ (%)	*N* pos (%)	*N* pos (%)	*N* pos (%)	*N* equ (%)
(T5 (0–150)	99	99 (100)	89 (89.9)	1 (1.0)	81 (81.8)	87 (87.9)	35 (35.4)	16 (16.2)
T6 (151–300)	45	45 (100)	39 (86.7)	0 (0)	33 (73.3)	38 (84.4)	11 (24.4)	7 (15.6)
T7 (301–500)	25	25 (100)	21 (84.0)	0 (0)	15 (60.0)	18 (72.0)	6 (24)	2 (8.0)
TVac (Vaccination)	68	68 (100)	68 (100)		68 (100)	68 (100)	68 (100)	0 (0)
All samples	237	237 (100)	216 (91.1)	1 (0.4)	197 (83.1)	211 (89.0)	120 (50.6)	25 (10.5)
Positive agreement % ^a^		reference	91.6		reference	97.0	72.1	
95% CI			87.3–94.8			93.5–98.9	65.3–78.2	
Negative agreement %		reference			reference	50.0	92.5	
95% CI						33.8–66.2	79.6–98.4	

^a^ Calculation of agreements was based on the respective in-house tests, with equivocal results from EUROLINE Profile IgG and EUROIMMUN SARS-CoV-2 NeutraLISA counted as positive. Abbreviations: pos, positive; equ, equivocal; dpso/dpPCR, days post-symptom onset/days post-PCR; TVac, days post-vaccination.

## Data Availability

The data presented in this study are available on request from the corresponding author.

## Supplementary Materials

The following supporting information can be downloaded: https://www.mdpi.com/article/10.3390/v16010091/s1, Figure S1: Dynamics of SARS-CoV-2-specific IgG and NAbs during an acute infection. Tests used for Ab measurement are indicated on x-axes. The percentage of samples in which Ab was detected is depicted in black (positive) and in which Ab was not detected in grey (negative). Figure S2: Course of antibodies in patients (a) undergoing cancer treatment, (b) treated with immunosuppressives, or (c) receiving antibody therapy. The course of NAb is shown on the left plot, with NAbs determined by VNT plotted on the left *Y*-axis (black), by cPass plotted on the right *Y*-axis (purple), and by NeutraLISA plotted on the right *Y*-axis (green). Plots on the right side show IgG detected in the same samples with total IgG determined with IIFT (black), plotted on the left *Y*-axis, S1 IgG (green), S2 IgG (blue), and NP IgG (red), all determined with Profile IgG and plotted on the right *Y*-axis; Figure S3: Course of SARS-CoV-2-specific IgG antibodies determined with IIFT, or Profile IgG, in the sera of convalescent patients Total IgG (black) determined by IIFT plotted on the left *Y*-axis; IgG values for anti-S1 (green), anti-S2 (blue), and anti-NP (red) determined by Profile IgG plotted on the right *Y*-axis; Figure S4: Dynamics of SARS-CoV-2-specific IgG and NAbs in samples from convalescent patients Tests used for Ab measurement are indicated on x-axes. The percentage of samples with Ab presence is depicted in black (positive) and without Ab detection in gray (negative). Figure S5: Course of SARS-CoV-2-specific NAbs in sequential serum samples from convalescent patients. NAbs were determined with VNT (black) plotted on the left *Y*-axis, and inhibition rates were determined with cPass (purple) and NeutraLISA (green) plotted on the right *Y*-axis.
