# Supplementary figures and images for: Performance Analysis of Serodiagnostic Tests to Characterize the Incline and Decline of the Individual Humoral Immune Response in COVID-19 Patients: Impact on Diagnostic Management
Source: Viruses. 2024 Jan 6;16(1):91. doi: 10.3390/v16010091 (PMC10820597; doi:10.3390/v16010091)

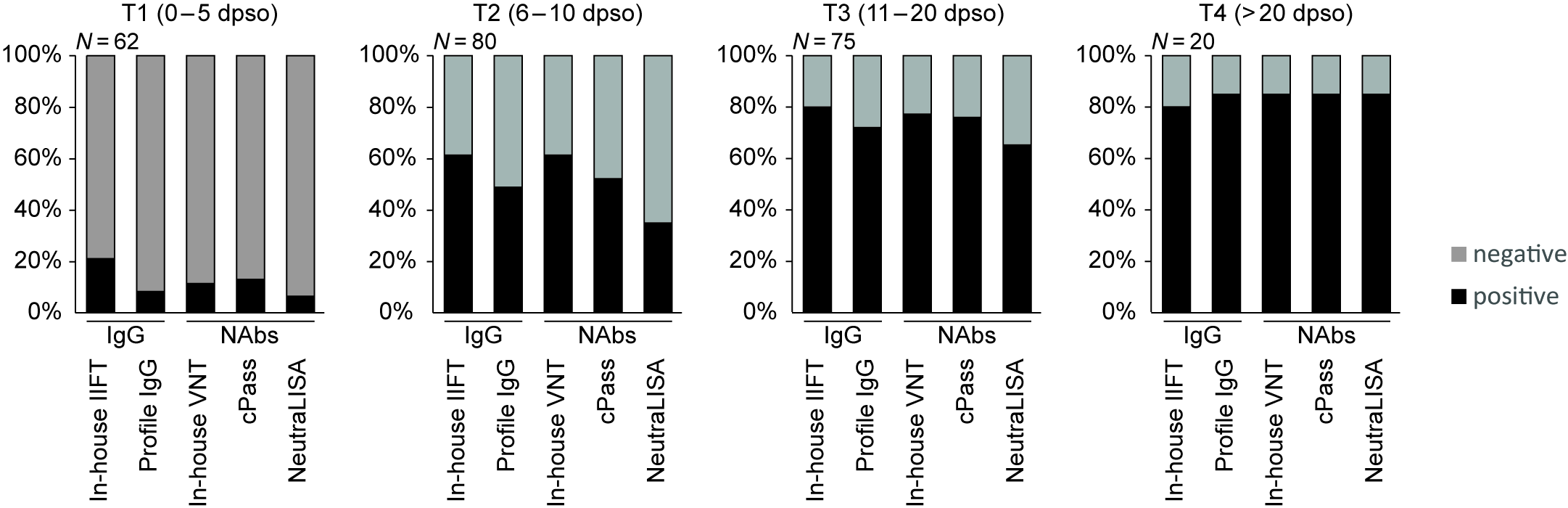

Supplement: Supplementary file 1 [file viruses-16-00091-s001.zip › 231219-Supplementary Figure 1.tif]

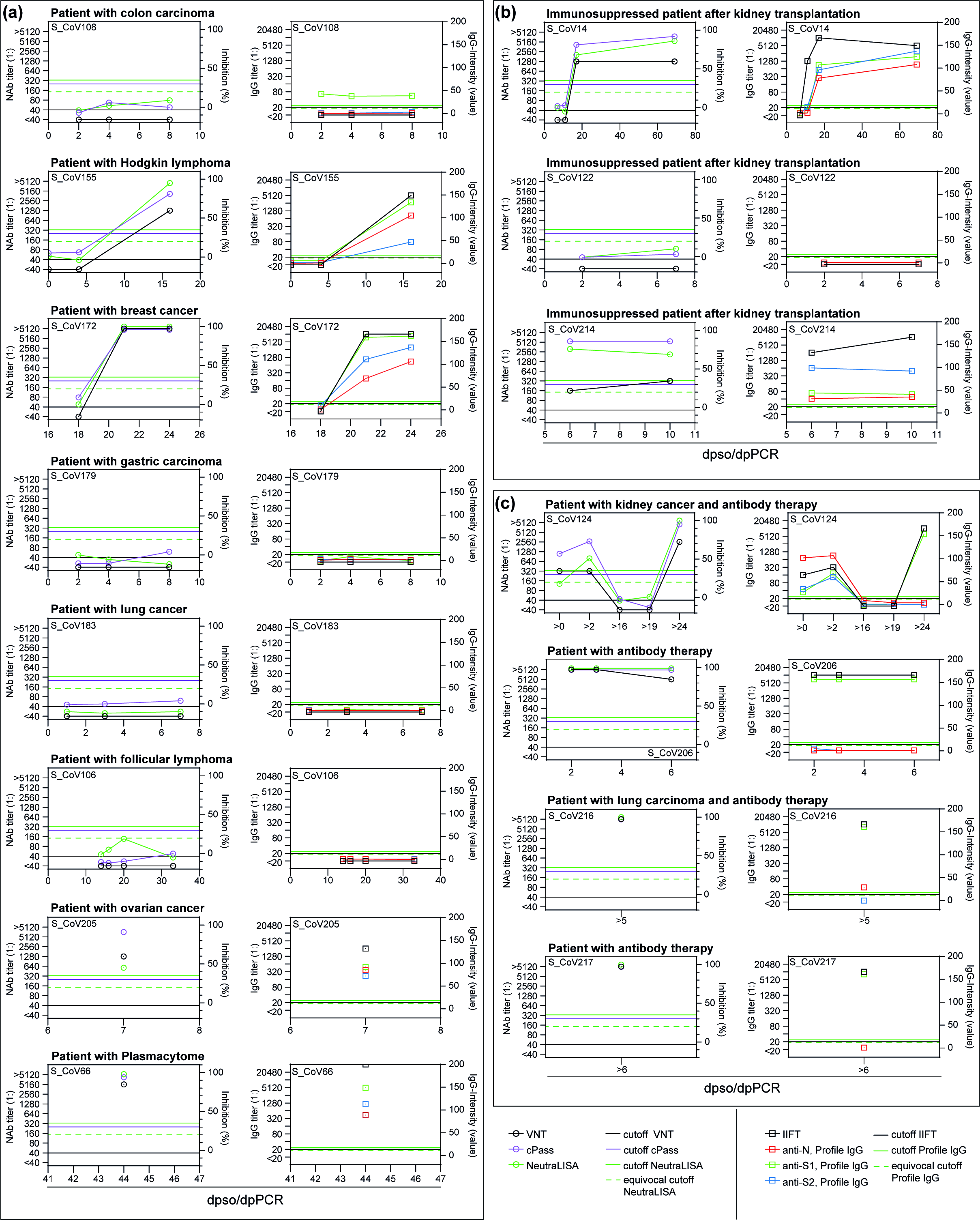

Supplement: Supplementary file 1 [file viruses-16-00091-s001.zip › 231219-supplementary figure 2.tif]

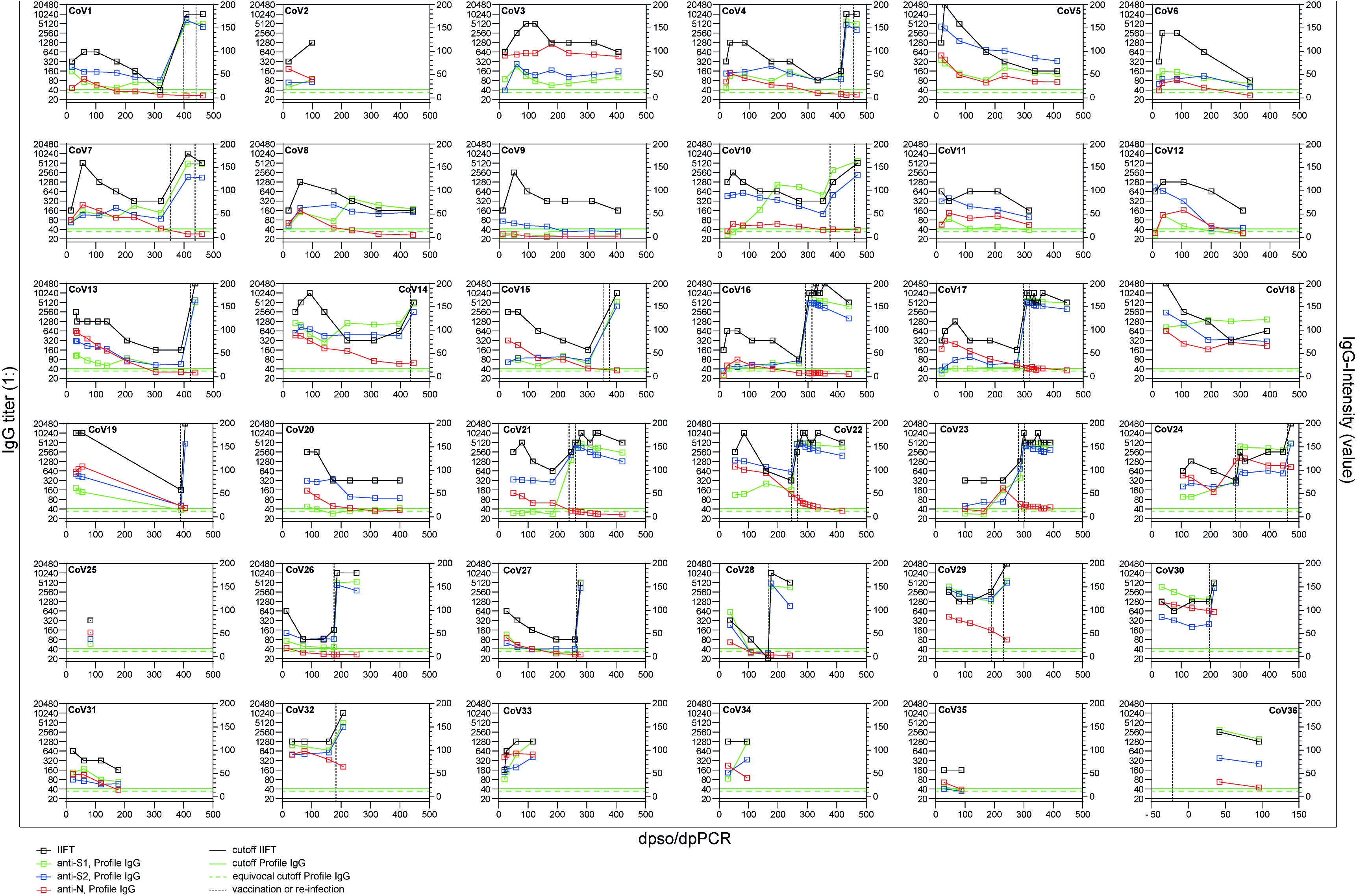

Supplement: Supplementary file 1 [file viruses-16-00091-s001.zip › 231219-Supplementary Figure 3.tif]

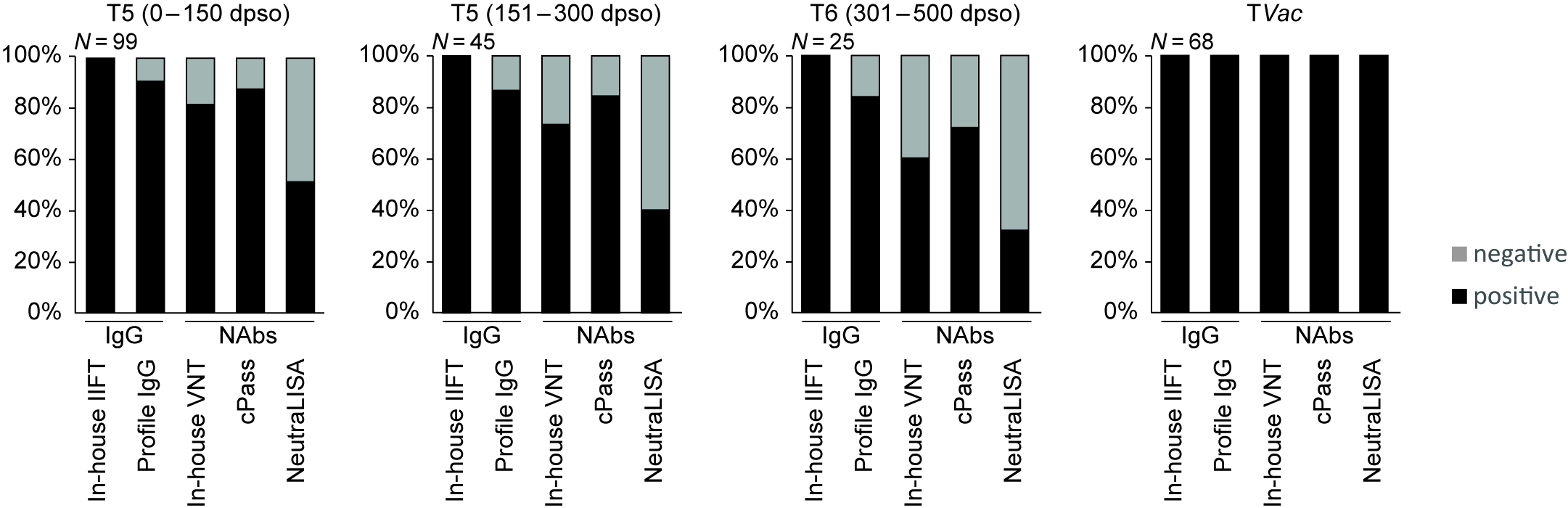

Supplement: Supplementary file 1 [file viruses-16-00091-s001.zip › 231219-Supplementary Figure 4.tif]

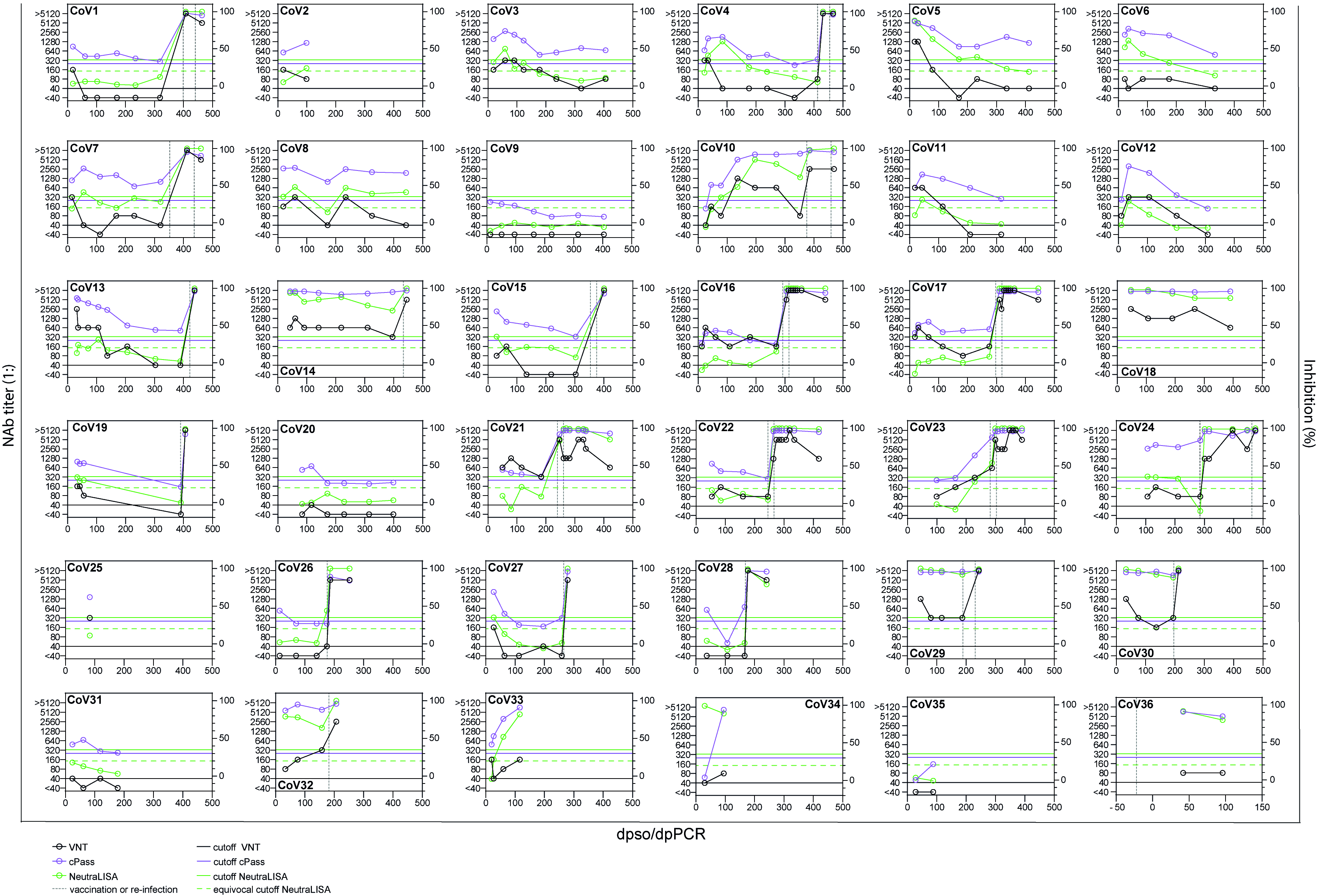

Supplement: Supplementary file 1 [file viruses-16-00091-s001.zip › 231219-supplementary figure 5.tif]
